# Effect of Nb_2_O_5_ doping on improving the thermo-mechanical stability of sealing interfaces for solid oxide fuel cells

**DOI:** 10.1038/s41598-017-05725-y

**Published:** 2017-07-13

**Authors:** Qi Zhang, Xinhang Du, Shengwei Tan, Dian Tang, Kongfa Chen, Teng Zhang

**Affiliations:** College of Materials Science and Engineering, Fuzhou University, Fuzhou, Fujian 350108 China

## Abstract

Nb_2_O_5_ is added to a borosilicate sealing system to improve the thermo-mechanical stability of the sealing interface between the glass and Fe-Cr metallic interconnect (Crofer 22APU) in solid oxide fuel cells (SOFCs). The thermo-mechanical stability of the glass/metal interface is evaluated experimentally as well as by using a finite element analysis (FEA) method. The sealing glass doped with 4 mol.% Nb_2_O_5_ shows the best thermo-mechanical stability, and the sealing couple of Crofer 22APU/glass/GDC (Gd_0.2_Ce_0.8_O_1.9_) remains intact after 50 thermal cycles. In addition, all sealing couples show good joining after being held at 750 °C for 1000 h. Moreover, the possible mechanism on the thermo-mechanical stability of sealing interface is investigated in terms of stress-based and energy-based perspectives.

## Introduction

Solid oxide fuel cells (SOFCs) are regarded as having great potential for fuel flexibility, high energy conversion efficiency and environmental friendliness^1^. Glass and glass ceramic sealing materials are most intensively used in planar SOFCs, and they are required to have desirable chemical and thermo-mechanical stability under typical operating conditions^2^. Especially, the thermo-mechanical stability is critical for the SOFC stacks since significant difference thermal expansion of glass and cell components can cause high residual stress as well as crack at the sealing interfacial, which consequently impairs the cell integrity^3, 4^.

However, there is a practical obstacle of developing reliable sealing glass because of the occurrence of interfacial reaction between the sealing glass and other components of SOFCs, leading to performance degradation of SOFC stacks^5^. In particular, the interaction between glass-ceramics and Fe-Cr alloy interconnect is of a great concern. The transport of Cr from the metallic interconnect to form a Cr_2_O_3_ surface layer contributes to the formation of chromates, such as BaCrO_4_ and SrCrO_4_. These chromates possess a coefficient of thermal expansion (CTE) of 18–20 × 10^−6^ · K^−1^ substantially higher than ~10.0–12.0 × 10^−6^ · K^−1^ that of the sealing glass, leading to fracturing of the glass/metal interface^6^. In general, the thickness of the reaction zone (or inter-diffusion zone) and the interfacial bond strength are an indicative of the thermo-mechanical stability at the glass/metal interface^7, 8^.

Previous studies have shown that strontium ions in an open glass network can react with the Cr_2_O_3_ layer of interconnect and contribute to the sealing failure^9^. Conversely, Nb_2_O_5_ falls within the intermediate class of glass-forming oxides. Part of Nb^5+^ ions exist in NbO_4_ tetrahedra as network formers, while the rest is present in NbO_6_ octahedra. The NbO_4_ will convert to NbO_6_ when the Nb_2_O_5_ content is greater than 8 mol.%, causing an increase in non-bridging oxygen^10^. On the other hand, the borosilicate glass with Bi_2_O_3_ shows excellent glass sintering ability for SOFC operating application^11^. The borosilicate glass system with Bi_2_O_3_ dopant also stabilizes the B-O network, which is beneficial for reducing the detrimental interfacial reaction between sealing glass and cathode^12^. Moreover, the B_2_O_3_-SiO_2_-Bi_2_O_3_ glass system with ZnO dopant inhibits the chemical interaction between glass and interconnect^13^. Therefore, appropriate amount of 8 mol.% Nb_2_O_5_ (2, 4 and 8 mol.%) is added to a B_2_O_3_-SiO_2_-Bi_2_O_3_ glass system to condense the glass network structure in this work. And the effect of Nb_2_O_5_ on the chemical and thermo-mechanical stability of the sealing glass/metal interface is clarified using finite element analysis (FEA) and thermal cycling experiments.

## Results and Discussion

### Mechanical properties

**Figure 1:**
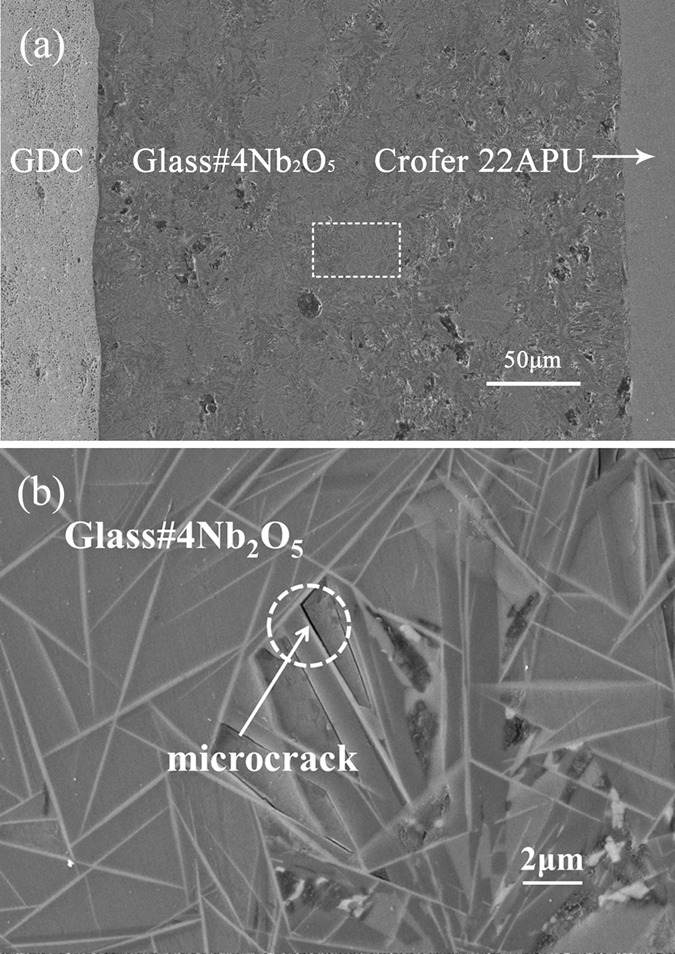
(**a**) Micrograph of the GDC/glass#4 Nb_2_O_5_/Crofer 22APU after 50 thermal cycles; (**b**) is a magnified image of the selected region in (**a**).

The interfaces of the sealing glass/cell components are required to withstand 70–200 kPa thermo-mechanical stress during cell operation^14^. Therefore, the resistance to thermal cycling can be considered as an indicator for evaluating the thermo-mechanical stability of the sealing interface^15^. The glass#4 Nb_2_O_5_ sample retains its good sealing ability after 50 thermal cycles (Fig. 1a), while fractures appear at the interface between Crofer 22APU and other glass. Microcracks are also observed between the needle-shaped phases (indicated by the arrows, Fig. 1b).

**Figure 2:**
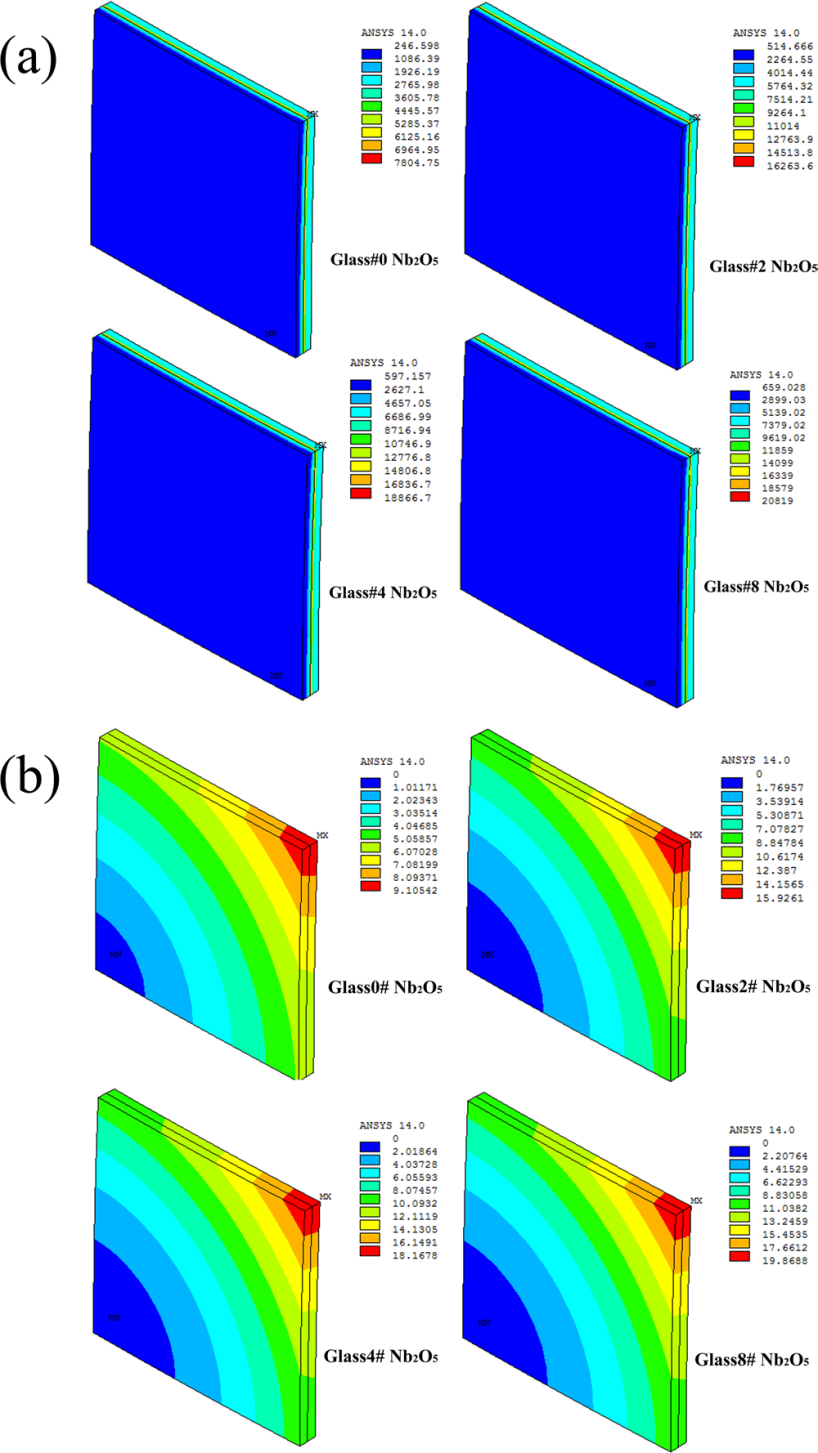
(**a**) The stress distribution and its magnitude at the glass/metal interface after thermal cycling for 50 times. (**b**) The displacement for the sealing couple between glass and Crofer 22APU after thermal cycling for 50 times.

To investigate the changes in thermal-mechanical properties of the sealing glass, FEA was performed on the distribution of thermal stress at the glass/metal interface after 50 thermal cycles, as shown in Fig. 2a. The maximum thermal stress between the sealing glass and Crofer 22APU after the 50 thermal cycles increases from 7805 MPa to 20819 MPa as the Nb_2_O_5_ content increases from 0 to 8 mol.%. High stress mainly distributes at the corners, and the maximum stress is observed in the glass#8 Nb_2_O_5_ sample due to its most distinctive CTE mismatch with the interconnect (~38%). The CTEs for the glass-ceramics glass#0 Nb_2_O_5_ to glass#8 Nb_2_O_5_ are 10.1 ± 0.1 × 10^−6^ · K^−1^, 9.1 ± 0.1 × 10^−6^ · K^−1^, 8.4 ± 0.1 × 10^−6^ · K^−1^, and 8.4 ± 0.1 × 10^−6^ · K^−1^, respectively. Figure 2b shows the displacements for the sealing couple of glass/interconnect after thermal cycling for 50 times. It is clear that the maximum displacement appears at the corner of sealing couple and the maximum displacement increases from 9.10542 mm to 19.8688 mm with the Nb_2_O_5_ content increasing. In brief, the crack forms initially at the corner of sealing couples and sealing couple of glass glass#8 Nb_2_O_5_ is easiest to fail according to the FEA analyst.

We also stimulated in the case of one thermal cycle using the FEA model. The maximum thermal stress due to CTE mismatch between the sealing glass and Crofer 22APU for the glass without Nb_2_O_5_ is 64 MPa, and increases to 133 MPa for the glass doped with 8 mol.% Nb_2_O_5_. These stress values are in the same magnitude to those reported in the literature. For example, Lin *et al*. investigated the thermal stress distribution due to CTE mismatch of the sealing glass-ceramics and metallic interconnect of SOFC stacks. The sample was cooled from 800 °C to room temperature, and then heated to 600 °C followed by start-up for cell operation. The maximum thermal stress of sealing glass-ceramics after one thermal cycle is about 20~100 MPa^16, 17^. Jiang *et al*. also reported the maximum thermal stress for the sealing glass-ceramics is about 80 MPa at 754 °C and 60~80 MPa at 854 °C^18^.

**Figure 3:**
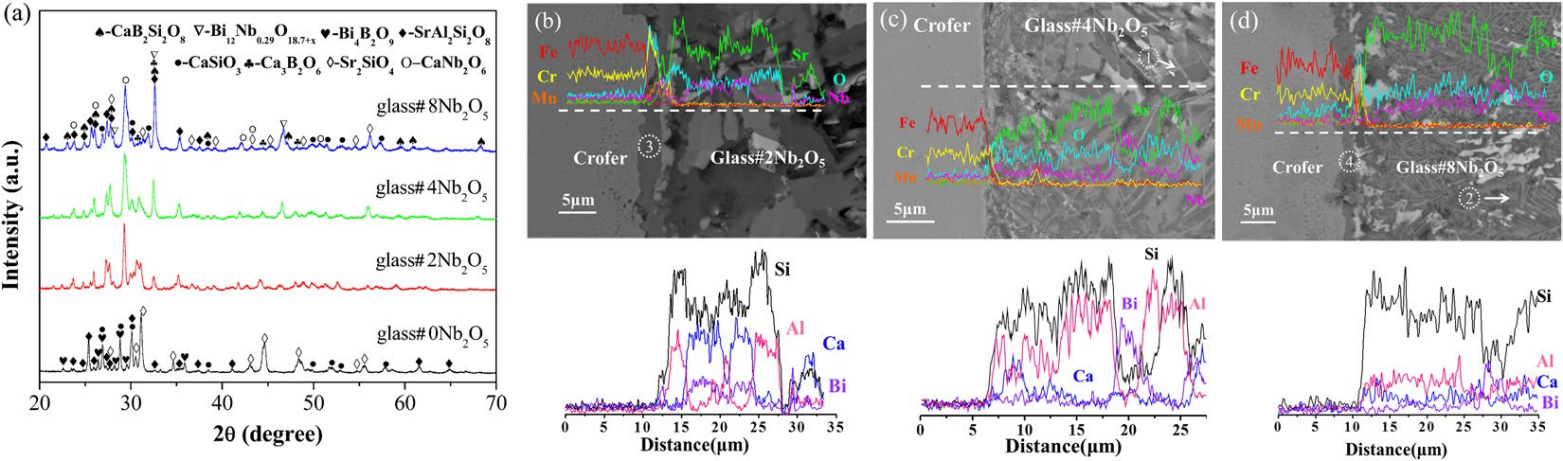
(**a**) XRD patterns of glass-ceramics held at 750 °C for 1000 h. SEM images and EDS elemental line scans of glass-metal interfaces held at 750 °C for 1000 h. (**b**)glass#2 Nb_2_O_5_, (**c**) glass#4 Nb_2_O_5_, and (**d**) glass#8 Nb_2_O_5_.

Figure 3a shows the XRD patterns of glass-ceramics held at 750 °C for 1000 h. Crystalline phases including SrAl_2_Si_2_O_8_ (JCPDS card no. 70–1862), CaSiO_3_ (JCPDS card no. 27–0088), Bi_4_B_2_O_9_ (JCPDS card no. 25–1089) and Sr_2_SiO_4_ (JCPDS card no. 38–0271) appear in the glass without Nb_2_O_5_, whereas SrAl_2_Si_2_O_8_, CaSiO_3_, Sr_2_SiO_4_, CaNb_2_O_6_ (JCPDS card no. 11–0619), Bi_1.7_Nb_0.3_O_3.3_ (JCPDS card no. 33–0210), Ca_3_B_2_O_6_ (JCPDS card no. 48–1885) and CaB_2_Si_2_O_8_ (JCPDS card no. 72–2298) are present in the Nb_2_O_5_ doped glass.

Shown in Fig. 3b to ﻿d﻿ are the SEM images and EDS profiles at the glass/metal interface held at 750 °C for 1000 h. The EDS results are summarized in Table 1. Significant amounts of Ca and Nb are detected in the needle phase regions (point#1 in Fig. 3c and point#2 in Fig. 3d). In addition, the number of needle phases increases with increasing Nb_2_O_5_ content. The CaNb_2_O_6_ phase can be characterized with various morphologies including microneedles or ellipsoid-like shapes, depending on the processing conditions^19, 20^. This confirms the formation of a CaNb_2_O_6_ phase in Nb_2_O_5_-doped glass-ceramics, in agreement with the XRD results. It has been reported that the CaNb_2_O_6_ phase shows a low thermal expansion (α_a_ = 2.80 × 10^−6^ K^−1^), which explains the fact that the CTE decreases with the increase of Nb_2_O_5_^21^.

**Table 1:** Quantitative EDS results in Fig. 3 (in at. %).

Spot	Al	Si	Ca	Sr	Nb	Bi	Fe	Cr	Mn
#1	12	23	22	21	18	4	—	—	—
#2	5	30	34	16	14	1	—	—	—
#3	2	1	0	1	2	2	2	60	29
#4	2	8	6	10	11	2	2	37	22

Moreover, some microcracks are formed surrounding the needle phases (CaNb_2_O_6_) in glass#4 Nb_2_O_5_ (see Fig. 1b). Residual stresses can be released upon the formation of microcracks, which increases the fracture resistance of glass^22–24^. Thus, the microcracks around the needle-shaped CaNb_2_O_6_ crystals are also beneficial to release the residual stress at the interface.

### Interfacial reaction

**Figure 4:**
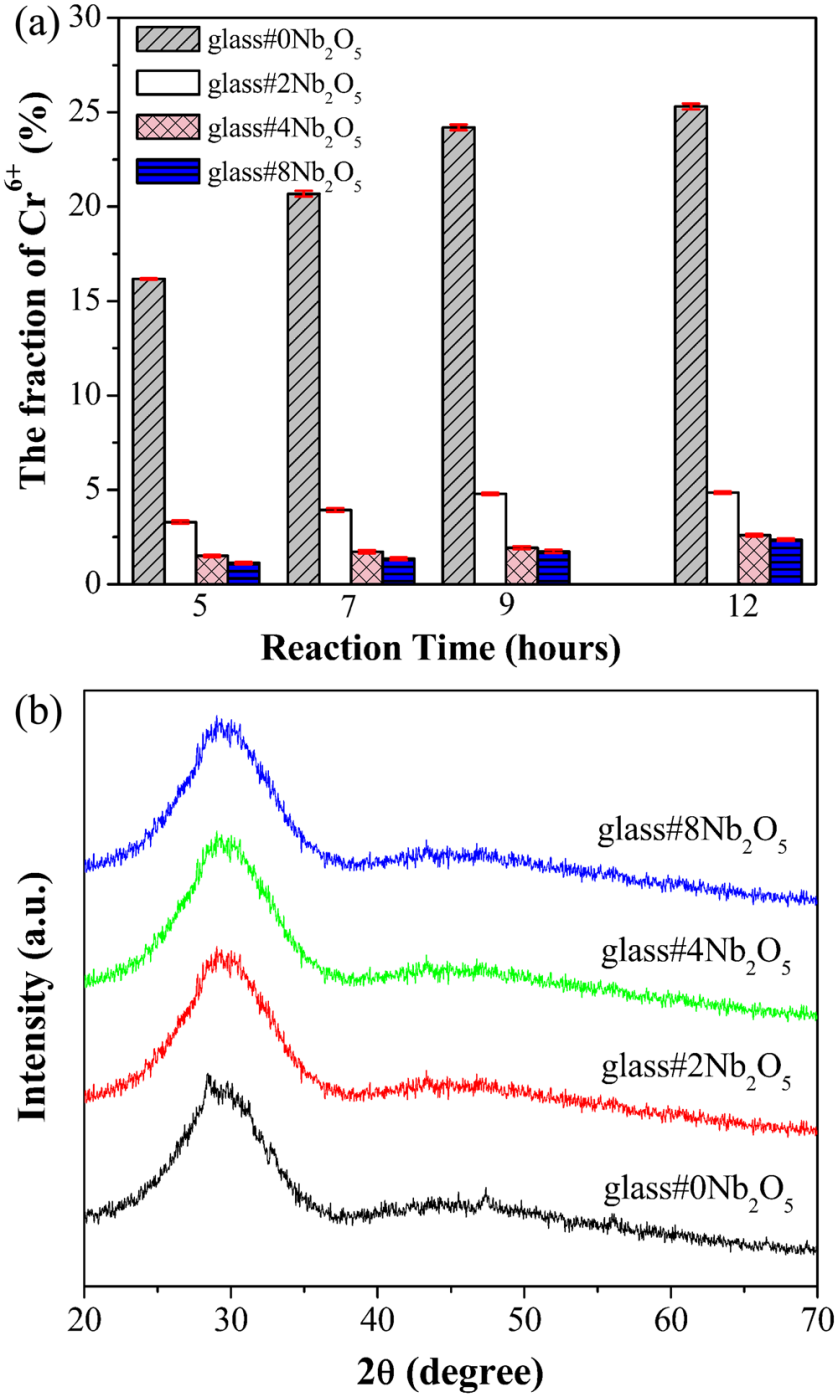
(**a**) Quantitative results of the Cr_2_O_3_/glass reaction couples held in air at 650 °C. (**b**) XRD patterns of the glass held at 650 °C for 20 h.

The formation of chromate phases is often observed at the glass/metal interface in the air side of SOFCs^25^. Figure 4a shows the quantitative analysis of the Cr_2_O_3_/glass reaction couples held in air at 650 °C. The reaction decreases with increasing the Nb_2_O_5_ content. As demonstrated previously, the mobility of Sr^2+^ ions in the glass is reduced due to condensation of Sr in the glass network structure, which in turn reduces their reactivity with Cr^26^. Therefore, the improved chemical compatibility between the sealing glass and Cr_2_O_3_ is most likely due to the condensed glass structure by the Nb doping, as the glass remains amorphous at 650 °C (Fig. 4b).

**Figure 5:**
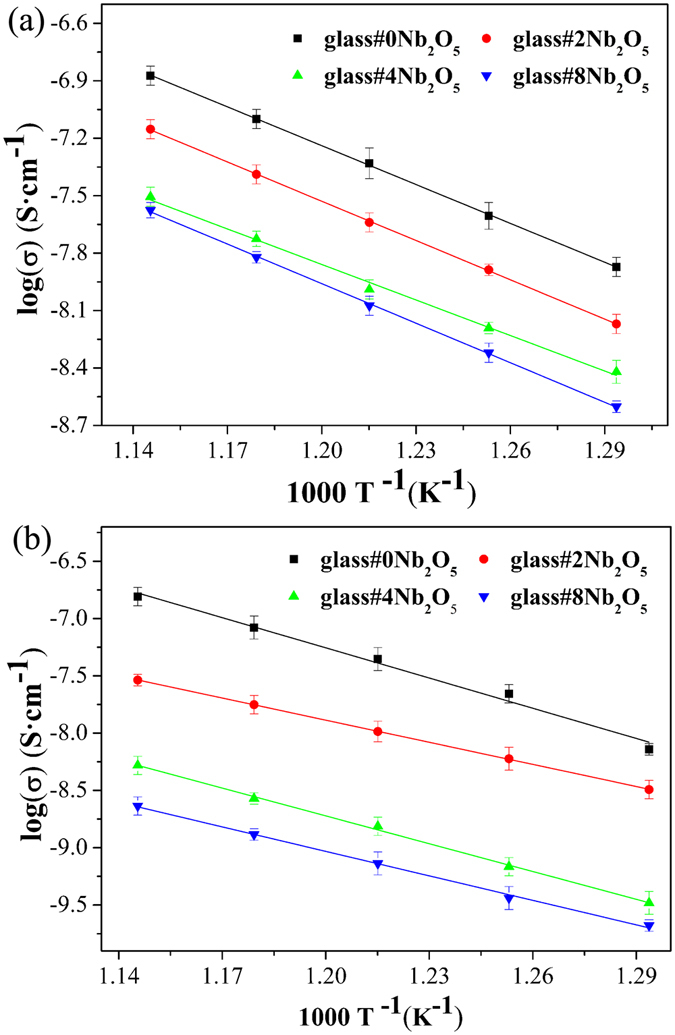
The temperature dependence of conductivity (log σ versus 1000 T^−1^), measured in air from 500 to 600 °C, for (**a**) as quenched glass and (**b**) glass-ceramics.

Table 2 shows the thermal and mechanical properties of glass and glass-ceramics. Some previous articles have reported that the CTE, glass transition temperature (T_g_) and glass softening temperature (T_d_) are intimately associated with the connectivity of the glass network. The increase in non-bridging oxygen and decrease in glass network connectivity will result in the decrease in T_g_ and T_d_ as well as increase in CTE^27, 28^. Thus, in this work, the changes of T_g_, T_d_ and CTE of quenched glass indicate the condensed network structure of sealing glass by Nb_2_O_5_ doping. Similarly, it has been reported that more non-bridging oxygens in glass network reduces the connectivity of glass network and decreases the density of glass. The hardness of glass also increases as the glass network becomes rigid^29–32^. Hence, the increase in density and Vickers hardness of glass also confirms the strengthened glass network in present work. Figure 5 shows the temperature dependent conductivity plots (log σ versus 1000 T^−1^) for as quenched glass and glass-ceramics, measured in air from 500 to 600 °C. The conductivity of glass and glass-ceramics meets the insulating requirement of sealing glass (<10^−4^ S cm^−1^) for SOFCs application^33^. The decrease in conductivity of glass with increasing Nb_2_O_5_ content further confirms the densification of glass network by Nb_2_O_5_ doping in present work.

**Table 2:** Thermal and mechanical properties of glass and glass-ceramics.

Sample ID	glass#0 Nb_2_O_5_	glass#2 Nb_2_O_5_	glass#4 Nb_2_O_5_	glass#8 Nb_2_O_5_
**CTE(×10** ^**−6**^ **K** ^**−1**^, **200–600** **°C)**				
glass	12.2 ± 0.1	11.1 ± 0.1	10.6 ± 0.1	10.1 ± 0.1
glass-ceramics	10.1 ± 0.1	9.1 ± 0.1	8.7 ± 0.1	8.4 ± 0.1
**Measured by dilatometer(°C)**				
T_g_	669 ± 5	684 ± 5	718 ± 5	726 ± 5
T_d_	722 ± 5	751 ± 5	779 ± 5	782 ± 5
**Density(g cm** ^**−3**^ **)**				
glass	3.55 ± 0.01	3.56 ± 0.01	3.68 ± 0.01	3.83 ± 0.01
glass-ceramics 750 °C for 1000 h	3.31 ± 0.01	3.42 ± 0.01	3.48 ± 0.01	3.69 ± 0.01
**Vickers hardness(GPa)**				
glass	11.69 ± 0.01	12.98 ± 0.01	15.97 ± 0.01	16.48 ± 0.01

The results of interfacial reaction between glass and metallic interconnect is consistent with changes in thermal, mechanical and electrical properties of the glass material with Nb_2_O_5_ doping.

In addition, the thickness of the interfacial reaction zone decreases from ~4 µm to ~2 µm with increasing Nb_2_O_5_ (Fig. 3b–d). The detected Cr contents also decrease from 60 at.% to 37 at.% at the interface (point#3 in Fig. 3b and point#4 in Fig. 3d) with increasing Nb_2_O_5_ content. This indicates that the addition of Nb_2_O_5_ significantly improves the chemical compatibility between sealing glass and Fe-Cr interconnect.

Fracturing occurs at the interface between the interconnect and the reaction zone (not shown here). Müller *et al*. suggested that the sealing glass should be made as thin as possible, since the maximum energy release rate increases significantly with the increase in the thickness of sealing glass in an interconnect/sealing glass/interconnect diffusion couple^34^. Regarding the reaction zone as a thin layer, the thinner reaction zone with Nb_2_O_5_ dopant should decrease the maximum energy release rate and improve the resistance to crack initiation.

There are two criteria including stress and energy for predicting crack initiation at the interface^35^. Fracturing will be observed at an interface if the tensile stress is greater than the interface strength, whereas crack initiation will occur at the interface when the energy release rate exceeds the threshold value. Leguillon *et al*. suggested that these two criteria must be considered together to provide a sufficient explanation of fracture conditions^36^. In this work, the CTE mismatch between glass and interconnect increases with Nb_2_O_5_ amount, leading to increased thermal stress. This implies that fracturing is more likely to occur with increasing the Nb_2_O_5_ amount according to the strength criterion. On the other hand, Nb_2_O_5_ reduces the thickness of the reaction zone, which decreases the maximum energy release rate and thus improves the resistance to crack initiation. Therefore, the sealing couple of Crofer 22APU/glass#4 Nb_2_O_5_/GDC shows the best thermal-mechanical stability against the thermal cycling. Moreover, all sealing couples after heat-treatment at 750 °C for 1000 h showed good joining between the glass and the GDC electrolyte.

**Figure 6:**
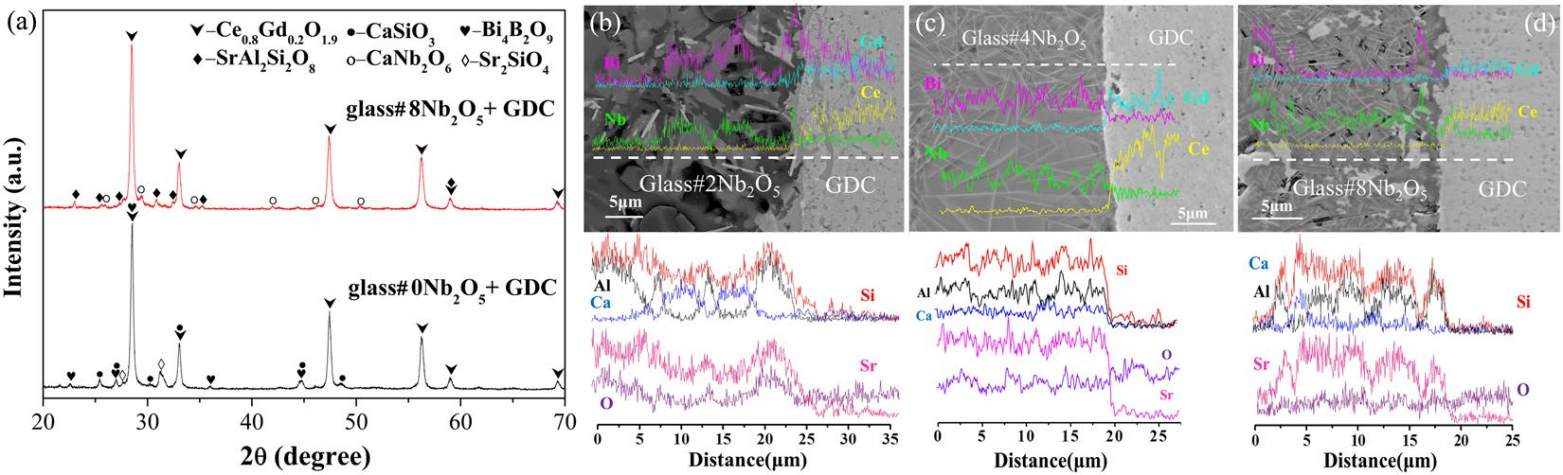
(**a**) XRD patterns of glass/GDC powder reaction couples held at 750 °C for 1000 h in air. Micrographs and elemental EDS line scans of the glass/GDC sealing couples held at 750 °C for 1000 h, for (**b**) glass#2 Nb_2_O_5_, (**c**) glass#4 Nb_2_O_5_ and (**d**) glass#8 Nb_2_O_5_.

Figure 6a shows the XRD patterns of glass-GDC powder couples held at 750 °C for 1000 h in air. The main phase in the glass and GDC reaction couples (50:50 w/w) is Ce_0.8_Gd_0.2_O_1.9_. This implies that the glass is chemically compatible with the GDC electrolyte. On the other hand, there is observation of cation interdiffusion at the glass/GDC interface. Figure 6b to d show the micrographs and EDS line scans of the glass/GDC sealing couples held at 750 °C for 1000 h, for glass#2 Nb_2_O_5_, glass#4 Nb_2_O_5_ and glass#8 Nb_2_O_5_. The EDS line scan reveals that the interdiffusion zone is reduced from ~3 μm to ~1 μm with increasing the Nb_2_O_5_ content. This indicates that the Nb_2_O_5_ dopant enhances the chemical compatibility between the sealing glass and GDC.

## Conclusions

In this work, the effect of Nb_2_O_5_ doping on the thermal-mechanical stability of sealing interfaces is investigated experimentally as well as using FEA simulations. The doping of Nb_2_O_5_ increases the CTE mismatch between the glass and the interconnect, leading to increased thermal stress. The Nb_2_O_5_ doping also condenses the glass network, reducing the interdiffusion between the glass and the interconnect. This decreases the maximum energy release rate, thereby improving the resistance to crack initiation. Moreover, some microcracks are form around the needle-shaped CaNb_2_O_6_ crystals. The presence of microcracks is beneficial to release the residual stress. The insights into the improved thermo-mechanical stability of sealing interfaces by proper addition of Nb_2_O_5_ will facilitate the development of reliable sealing materials.

## Method

### Preparation of glass

The base glass with nominal molar composition of 24.0CaO-24.0SrO-8.0B_2_O_3_-7.0Al_2_O_3_-35.0SiO_2_-2.0Bi_2_O_3_ was melted at 1350 °C for 1 h in a platinum crucible, named glass#0 Nb_2_O_5_. The melt was immersed in water for quenching and the glass powder was crushed and sieved to a particle size range of 45–53 µm. The glass with Nb_2_O_5_ (2, 4, 8 mol.%) doped to the base glass was also prepared as above, and named glass#2 Nb_2_O_5_, glass#4 Nb_2_O_5_, glass#8 Nb_2_O_5_ respectively. Some glass powders were held at 650 °C for 20 h and 750 °C for 1000 h in air. The crystalline phases in the samples were identified using X-ray diffraction (XDS 2000, Scintag, Inc.) with a monochromator Cu Kα radiation (k = 1.54 Å).

### Thermal cycling stability and finite element analysis

The Crofer 22APU/glass/Gd_0.2_Ce_0.8_O_1.9_ (Sinopharm Chemical Reagent Co., Ltd.) samples were prepared using a slurry technique, and the sandwiched samples were subjected to thermal cycles for 50 times after heat-treatment at 750 °C for 1000 h in air. A thermal cycle consists of heating the sealing couple from ambient temperature to 750 °C in 30 min, and then cooling in air in another 30 min. The micrographs of the sealing interface after 50 thermal cycles and after polishing were characterized by field emission scanning electron microscopy (FE-SEM; Supra-55, Zeiss, Inc.). FEA was selected to simulate the thermal stress distribution and the displacement caused by the CTE mismatch between different SOFC components in a uniform temperature field, in order to evaluate the thermo-chemical stability of the glass/metal interface.

### Characterization of thermal properties of glass and glass-ceramics

The glass transition temperature (T_g_), softening temperature (T_d_), and the CTE (at 200–600 °C) of quenched glass and species held at 750 °C for 1000 h (referred to ‘glass-ceramics’) was obtained using a dilatometer (DIL402C, NETZSCH, Inc.) at a heating rate of 10 °C · min^−1^ in air.

The density of the glass was measured using the Archimedes method, with deionized water as the liquid medium. The Vickers hardness of the glass was measured by a Vickers indentation method, using a HMV-2000 Micro Hardness Tester (Shimadzu, Japan) with a load of 0.5 N for 10 s. The electrical conductivity of the glass and glass-ceramics was measured in air from 600 to 700 °C by a high resistance meter (4339B, Agilent, Inc.).

### Characterization of interfacial reaction

A mixture of 10 wt.% Cr_2_O_3_ powder and 90 wt.% glass powder was reacted at 650 °C in air, to keep glass in amorphous state. The UV-Vis absorption spectra of the reaction products in aqueous solution were recorded using an Optima 2000 DV (Perkin Elmer, Inc.). The detailed procedure of this interfacial reaction has been described previously^37^.

The glass was bonded to Crofer 22APU and GDC substrates and held at 750 °C for 1000 h. Micrographs of the sealing interface were obtained using SEM equipped with energy dispersive spectroscopy (EDS; X-Max, OXFORD instruments, Inc.).

Kaur *et al*. reported that the thermal stress is dependent on the thickness of sealing glass, and the deformation due to thermal stress decreases as the thickness of the sealing glass increases from 0.25 to 1.5 mm^38^. Therefore, the thickness of the sealing glass in this study was kept constant (~250 µm) in all samples as well as in the FEA model.

A ~1 g mixture of glass and Gd_0.2_Ce_0.8_O_1.9_ powders (50:50, w/w) was reacted in air at 750 °C for up to 1000 h. The crystalline phases in the glass/GDC reaction couples were also analyzed by XRD. The sealing interfaces between glass and GDC were also investigated by SEM.
